# The application of fluorescein sodium for the resection of medulloblastoma

**DOI:** 10.1007/s11060-022-04035-2

**Published:** 2022-06-03

**Authors:** Zheng-he Chen, Xiang-heng Zhang, Fu-hua Lin, Chang Li, Jie-tian Jin, Zhi-huan Zhou, Si-han Zhu, Zhu-qing Cheng, Sheng Zhong, Zhen-qiang He, Hao Duan, Xia Wen, Jian Wang, Yong-gao Mou

**Affiliations:** 1 Department of Neurosurgery, Sun Yat-Sen University Cancer Center, 651 Dongfeng Road East, Yuexiu District, Guangzhou, 510060 People’s Republic of China; 2 State Key Laboratory of Oncology in South China, Guangzhou, 510060 People’s Republic of China; 3 Collaborative Innovation Center for Cancer Medicine, Guangzhou, 510060 People’s Republic of China; 4 Department of Pathology, Sun Yat-Sen University Cancer Center, Guangzhou, 510060 People’s Republic of China

**Keywords:** Fluorescein sodium, Medulloblastoma, Tumor visualization, Resection

## Abstract

**Introduction:**

Surgical resection of medulloblastoma (MB) remains a challenge. At present, a variety of tracers have been used for intraoperative tumor visualization. However, there are few reports on the intraoperative visualization of MB. Hence, we reported our experience of applying fluorescein sodium (FS) in MB surgery.

**Methods:**

We retrospectively analyzed the clinical information of patients with MB confirmed by surgery and pathology from January 2016 to December 2020 from Sun Yat-sen University Cancer Center. A total of 62 patients were enrolled, of which 27 received intraoperative FS and 35 did not. The intraoperative dose of FS was 3 mg/kg.

**Results:**

Among the 62 patients, 42 were males, and twenty were females. The age of onset in the FS group was 9.588 ± 7.322, which in the non-fluorescein sodium group was 13.469 ± 10.968, p = 0.198. We did not find significant differences in tumor location, tumor size, tumor resection, tumor histology, and preoperative symptoms (hydrocephalus, headache, vomit, balance disorder) between the groups. There was no significant difference in the postoperative symptoms (hydrocephalus, headache, vomiting, balance disorder, and cerebellar mutism). However, patients in the FS group had a relatively low incidence of balance disorder and cerebellar mutism. There was definite fluorescence of tumor in all cases of the FS group, and even the tiny metastatic lesion was visible. No case had side effects related to the use of FS.

**Conclusions:**

FS is safe and effective in MB surgery. Whether the application of FS for surgery can reduce complications remains to be studied in the future.

## Introduction

Medulloblastoma (MB) is the most common malignancy in childhood brain tumors. The current management strategies for this disease are maximal surgical resection followed by craniospinal irradiation and adjuvant chemotherapy. Despite this multipronged approach to therapy, nearly one-third of MB patients die from this disease [1]. Patients of MB have an increased risk of poor quality of life due to the severe adverse effects of MB and its management. Although researchers found that gene mutations dominate the final fate of this disease, surgery still plays a critical role in the management of MB. The extent of surgical resection remains a prognostic factor. Patients > 3 years of age who underwent a gross total resection (GTR) or near total resection (NTR; < 1.5cm^2^ residual tumor) have better outcomes compared with those who underwent subtotal resection (STR) [2, 3]. But trying to reach GTR or NTR goal may cause disastrous complications, such as impaired speech, cognition, and motor function. Intra-operative neurophysiological monitoring reminds surgeons of the existence of neural tracts in the brain stem, however, differentiating the precise border of the tumor is still a challenge.

Tumor visualization is a potential technology to benefit the resection of tumors. Several agents have demonstrated their value in various kinds of malignancy. 5-aminolevulinic acid (5-ALA), a natural biochemical precursor of hemoglobin that improves the synthesis and concentration of fluorescent porphyrins in various tumors, is used in EURO and USA [4, 5]. However, due to the high cost of 5-ALA, Fluorescein sodium (FS) is an alternative fluorophore, which is inexpensive and biosafe. Studies reveal FS can strongly stain the contrast enhancement regions in high grade gliomas (HGGs) [6–8], and benefit the sampling of low grade glioma [9]. But there is only one case report depicting the application of FS in the surgical resection of MB [10].

Hence, we retrospectively analyzed our experiences with the application of FS in 27 MB patients and proposed that fluorescein is safe and effective in the resection of MB.

## Methods

###  Patients

Clinical, pre-, and post-operative gadolinium-enhanced magnetic resonance imaging (MRI), surgical and histopathological data of a total of 62 patients with the diagnosis of MB from January 2016 to December 2020 from Sun Yat-sen University Cancer Center were collected. The patients were dichotomized whether the FS was used or not. Among them, 27 patients underwent FS-guided operations. All patients had symptoms caused by neurologic deterioration. The patients were followed up by clinical examination and MRI every 3 to 6 months after surgery. The extent of tumor resection was evaluated by comparing the results of pre-operative and post-operative neuroimaging. The tumor size was evaluated by the Chang Staging Classification [11]. Written informed consent was obtained on behalf of the adult patients or child patients from parents.

###  Protocol for fluorescein sodium guided surgery

54 patients underwent a standard suboccipital craniotomy, achieving the goal of gross total resection for MB in the posterior fossa. Seven patients underwent a retrosigmoid approach, and 1 patient performed a supratentorial approach.

The standard FS guided operation was previously reported [12]. At induction, a predefined dose of 3 mg/kg of FS was injected intravenously via a central line prior to general anesthesia, after the negative FLS skin test was obtained. With planting the electrodes of intraoperative neuromonitoring, the patient was laid in the prone position, and the head was fixed in a Mayfield head-holder. No need for the maximum flexibility of the neck in case of focal intracranial hypertension. Operations were performed under white light using an operation microscope (neurosurgery microscope OPMI PENTERO 900, German Carl Zeiss Meditec AG) and the neurosurgeon repeatedly switched into blue illumination mode to visualize yellow fluorescence in the operative field. Neurophysiological monitoring techniques, such as Somatosensory Evoked Potentials (SSEP), Motor Evoked Potentials (MEP), and Brainstem Auditory Evoked Potentials (BAEP), were applied.

After identifying the marginal zone of tumor invasion under the yellow fluorescence field, the operation proceeded while the microscope was switched into the white light mode. With surgery proceeding, the surgeon could switch to a different light mode to meet the demand of surgical requirements.

### Statistical analyses

Nominal variables were analyzed using a two-tailed Fisher's exact test or chi-square test followed by post hoc two-tailed Fisher's exact tests when applicable. Ordinal and continuous variables were analyzed with the Mann–Whitney U test. P-values with corresponding odds ratios (OR) were presented in the tables. P < 0.05 was considered statistically significant. Tests were performed using R software version 3.6.2 and SPSS software (v24.0).

#### Univariant and multivariant analyses

To assess the factors that are related to the OS of the patients, univariant and multivariant analyses were performed between the FS and non-FS subgroups by integrating clinical variables including the age, gender, tumor histology, tumor location, type of resection, presence of pre-op hydrocephalus, headache, vomit, etc. Variables that were observed with significant differences between FS and non-FS subgroups in the univariate analysis were selected for multivariate analysis.

#### Survival analysis

The p-value for survival analysis was calculated using R packages “survival” and “surveminer” based on the overall survival (OS) of the patients [13]. Kaplan–Meier survival (K–M) curves were plotted for FS and non-FS subgroups. Patients without information were excluded.

## Results

###  Descriptive parameters

Sixty-two consecutive patients, 42 males, and twenty females were diagnosed with MB. The patients' ages ranged from 1 year and 19 months to 48 years. The FS-guided operation proceeded in 27(43.5%) patients. Both the FS group and NFS group were comparable in terms of gender, age at surgery, pre-op hydrocephalus, headache, vomit, balance disorder, tumor location, tumor size, histology, and resection (Table 1). No significance was found.

**Table 1:** Clinical characteristics of patients with MB

	FS n = 27(%)	NFS n = 35(%)		P-value	OR
Gender				0.276	0.503
Male	16(59.3)		26(74.3)		
Female	11(40.7)		9(25.7)		
Age at surgery				0.198	
Mean	9.588		13.469		
SD	7.322		10.968		
Range	1.75–42		2–48		
Pre-op hydrocephalus				0.442	2.232
Yes	25(92.6)		28(80)		
No	2(7.4)		5(14.3)		
Unknown	0(0)		2(5.7)		
Headache				0.773	0.765
Yes	17(63)		25(71.4)		
No	8(29.6)		9(25.7)		
Unknown	2(7.4)		1(2.9)		
Vomit				0.785	1.295
Yes	19(70.4)		22(62.9)		
No	8(29.6)		12(34.3)		
Unknown	0(0)		1(2.9)		
Balance disorder				0.788	0.782
Yes	13(48.1)		18(51.4)		
No	12(44.4)		13(37.1)		
Unknown	2(7.4)		4(11.4)		
Tumor Location				0.237	
Vermis	24(88.9)		30(85.7)		
Hemisphere	3(11.1)		2(5.7)		
Bilateral	0(0)		3(8.6)		
Tumor size				0.651	
1	2(7.4)		4(11.4)		
2	14(51.9)		18(51.4)		
3	9(33.3)		11(31.4)		
4	2(7.4)		2(5.7)		
Histology				0.524	
Classic	19(70.4)		20(57.1)		
Desmoplastic ± nodularity	4(14.8)		6(17.1)		
Large-cell anaplastic	3(11.1)		4(11.4)		
Not otherwise specified	1(3.7)		5(14.3)		
Resection				0.988	
GTR	8(29.6)		10(28.6)		
NTR	9(33.3)		13(37.1)		
STR	10(37)		11(31.4)		
Biopsy	0(0)		1(2.9)		

*FS* fluorescein sodium, *GTR* gross total resection, *MB* medulloblastoma, *NFS* non-fluorescein sodium, *NTR* near total resection, *OR* odds ratio, *SD* standard deviation, *STR* subtotal resection

To identify whether the use of FS was a benefit to the alleviation of symptoms, we analyzed the appearance of post-op symptoms in the FS group and NFS group. Whether it was about “hydrocephalus”, “headache”, “vomit”, “balance disorder” or “cerebellar mutism”, we did not find significant statistical differences (Table 2). Regarding the two types of symptoms of “balance disorder” and “cerebellar mutism, the incidence in the FS group was lower than that of NFS. They are 7.4% vs. 20%, and 3.7% vs. 20%, respectively.

**Table 2:** The appearance of post-op symptoms of patients with MB

	FS n = 27(%)	NFS n = 35(%)	P-value	OR
Hydrocephalus			1	1.320
Yes	2(7.4)	2(5.7)		
No	25(92.6)	33(94.3)		
Headache			NA	NA
Yes	0(0)	0(0)		
No	27(100)	35(100)		
Vomit			1	1.794
Yes	0(0)	1(2.9)		
No	27(100)	34(97.1)		
Balance disorder			0.277	0.320
Yes	2(7.4)	7(20)		
No	25(92.6)	28(80)		
Cerebellar mutism			0.123	0.154
Yes	1(3.7)	7(20)		
No	26(96.3)	28(80)		

*FS* fluorescein sodium, *MB* medulloblastoma, *NA* not available, *NFS* non-fluorescein sodium, *OR* odds ratio

Univariate analysis of the clinical variables was performed (Table 3), and no statistically significant differences were observed, thus no variable was selected for multivariate analysis.

**Table 3:** Univariate analysis of the clinical variables

Variable	OR	95%CI	p-value
Gender (male/female)	1.986	0.675–5.842	0.213
Histology (classic/Des/LCA/NOS)	0.834	0.587–1.186	0.313
Location (vermis/hemisphere/bilateral)	1.704	0.538–5.4	0.365
Resection (GTR/NTR/STR/Biopsy)	1.017	0.555–1.864	0.956
Age at surgery			
(0–3/4–18/19–65)	2.320	0.714–7.537	0.162
Pre-op hydrocephalus	2.232	0.397–12.543	0.362
Headache	0.765	0.246–2.378	0.643
Vomit	1.295	0.438–3.834	0.640
Balance disorder	0.668	0.257–1.741	0.409
Tumor size			
(1/2/3/4)	0.844	0.429–1.664	0.625
KPS (< 70/ ≥ 70)	0.000	0–0	1.000
Recurrence	1.216	0.396–3.732	0.732

###  Fluorescence of MB

**Fig. 1:**
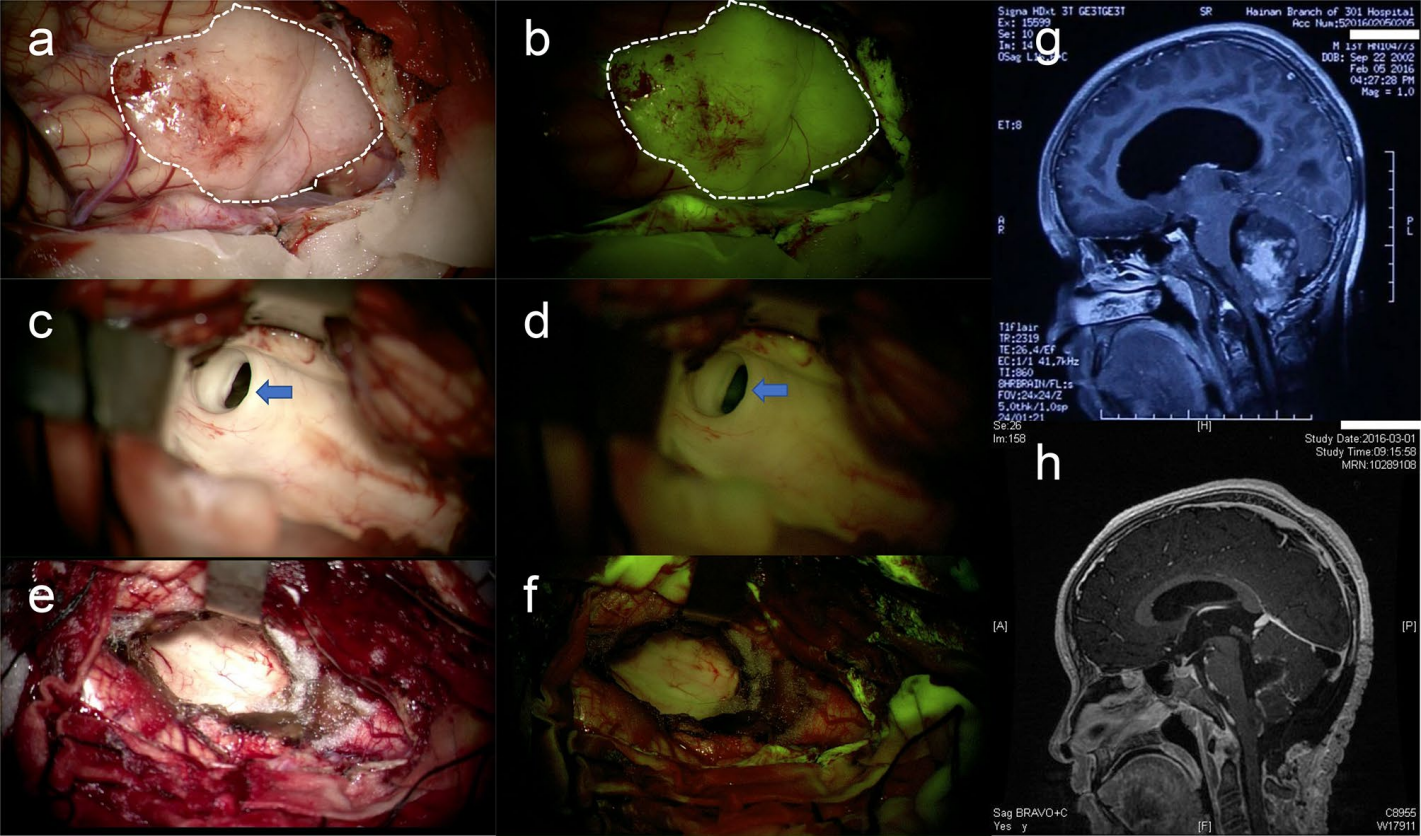
An 8-year-old boy underwent fluorescein sodium guided surgery for medulloblastoma of the cerebellar vermis. **a** The tumor (showed the white dashed frame) herniated from the cerebellomedullary fissure under the white light and (**b**) the yellow light. When the tumor was removed, the opening of the midbrain aqueduct (**c,** the white light mode; **d**, the yellow light mode) (blue arrow) and the fourth ventricle were revealed (**e**, the white light mode; **f**, the yellow light mode). **g**, **h** Sagittal magnetic resonance imaging shows complete removal of the tumor

**Fig. 2:**
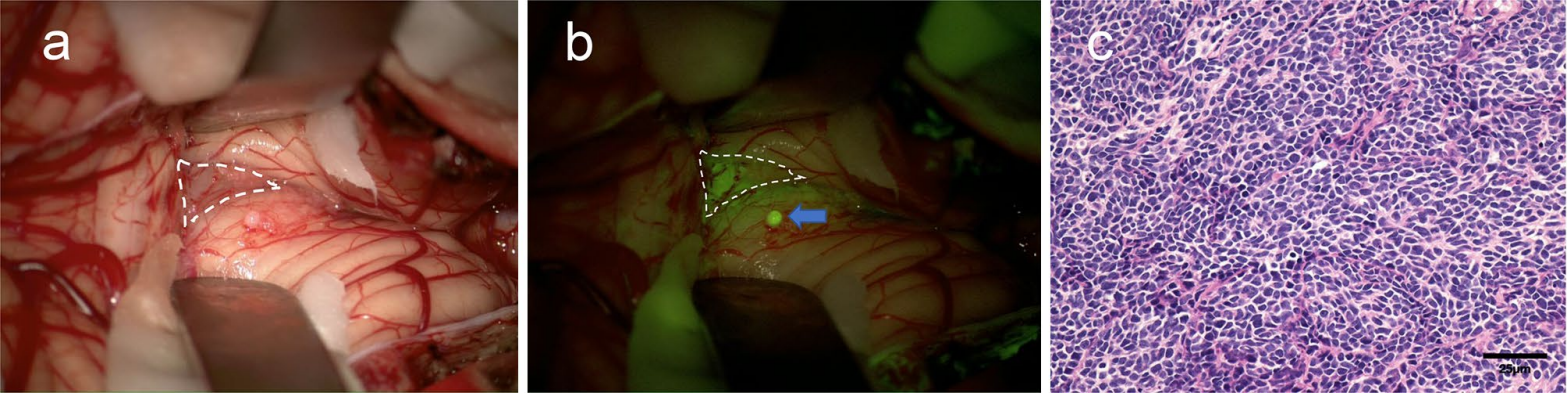
Illustration of the value of fluorescein sodium in the visualization of micrometastasis. A 7-year-old boy underwent surgery for medulloblastoma of the cerebellar vermis with the aid of fluorescein sodium. The tumor tissue was shown in the area of the white dashed (**a**, the white light mode; **b**, the yellow light mode). A tiny metastatic lesion (blue arrow) was found in the left cerebellar hemisphere under the yellow light mode and diagnosed as medulloblastoma with a feature of a dominant population of undifferentiated cells with a high nuclear-to-cytoplasmic ratio and mitotic (**c**)

In FS guided group, after a one-hour course of craniotomy, all tumors had a homogeneous and moderate yellow fluorescence under the yellow 560 nm filter (Fig. 1). When the tumor was removed, the compression of the Sylvius aqueduct was relieved, with yellow-tinged cerebral spinal fluid issued (Figs. 1 c, d). The fluorescence was very sensitive and even appeared in the tiny metastatic lesion less than 2 mm in diameter (Fig. 2).

The stable fluorescence of the tumor lasted nearly three hours on average. We did not observe any adverse effects, anaphylactic reactions, or postoperative neurological deterioration related to the fluorescein sodium-guided surgery.

###  Survival analysis for both groups

**Fig. 3:**
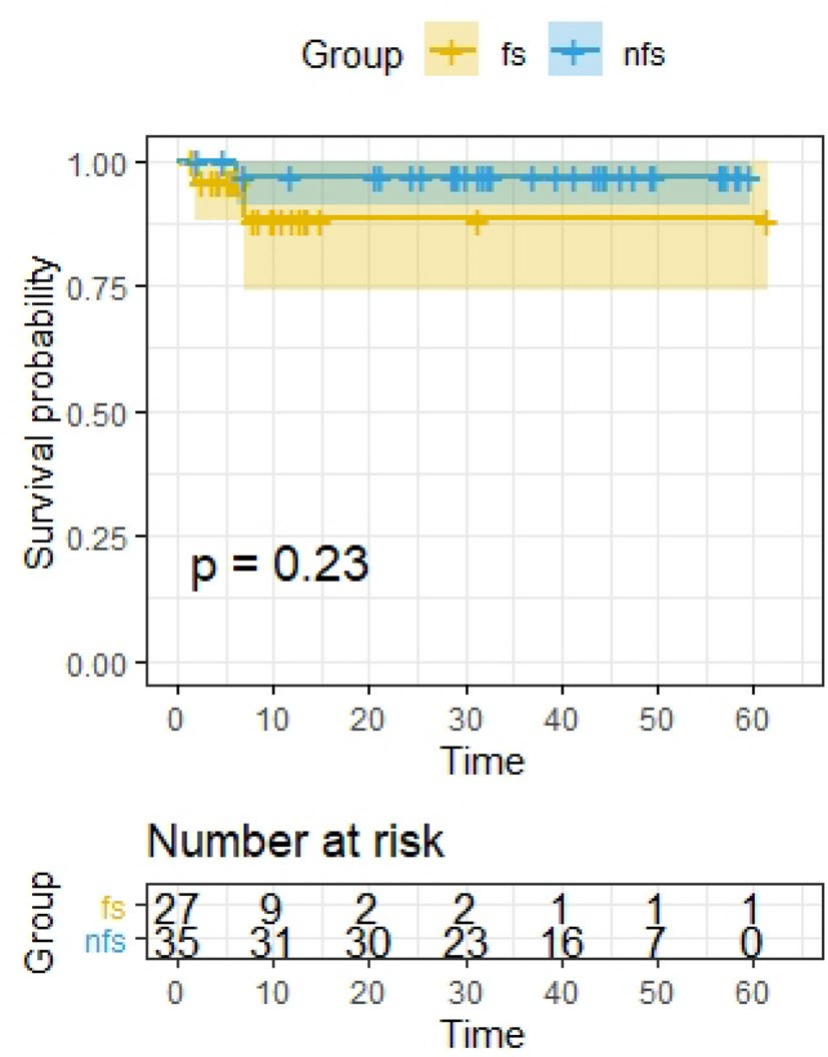
Survival analysis for both groups. No significant difference between the OS of FS and NFS was observed

Survival analysis for both groups has been made (Fig. 3). No significant difference between the OS of FS and NFS was observed.

## Discussion

Our retrospective study is the first report of a large sample of cases revealing the evaluation of fluorescein sodium guidance adjunct in the resection of medulloblastoma. We suggested that fluorescence can visualize the extent of the tumor, even the marginal metastatic lesion. No adverse side effects were found.

MB is the most common malignant brain tumor in children, and it can also affect a portion of the adult. It is an embryonal tumor of the cerebellum, derived from the discrete neuronal stem or progenitor cell. Formerly, MB has been classified into three major forms based on histology: classic, nodular/desmoplastic (ND), and large cell/anaplastic (LCA) [14]. In 2016, with the advances in genomics, the WHO consensus on the MB subgroup divided it into 4 major subgroups: WNT, SHH, Group 3, and Group 4 [15]. Despite progress in the diagnosis and therapies, one-third of MB causes the death of the patient [1]. The survivors usually suffer grievous clinical complications, such as cognitive deficits, cerebellar mutism, and secondary malignanc [16–18].

Surgery plays an important role in the standard management strategies for MB, achieving the sample for diagnosis, alleviating the mass effect, and rebuilding the CSF pathway. Albright et al. retrospectively reviewed 188 patients who underwent craniotomy for resection of MB, they concluded that the extent of residual tumor did correlate with good prognosis in certain children [2]. Thompson et al. made a retrospective multi-center integrated clinical and molecular analysis on the prognostic value of the extent of resection, in which 787 patients were enrolled. They reported a PFS benefit for GTR over STR (hazard ratio [HR] 1.45, 95% CI 1.07–1.96, p = 0.16) but no OS benefit (HR 1.23, 0.87–1.72, p = 0.24). No PFS or OS benefit for GTR or NTR was found [3]. Radical resection of the primary tumor is strongly encouraged at diagnosis, but not at the expense of causing permanent brain injury. GTR or NTR may cause disastrous complications. Intra-operative neurophysiological monitoring reminds surgeons of the existence of neural tract in the brain stem, however, differentiating the precise border of the tumor is still a challenge.

Tumor visualization is currently a method of surgical treatment. At present, the commonly used tumor imaging agents are 5-ALA, FS, and indocyanine green. Among them, there are still few reports on the application of tumor imaging agents for surgical removal of MB. Regarding 5-ALA, only 4 studies have been published so far. Two studies reported the application of 5-ALA in MB cell lines [19, 20]. Stummer et al. et al. surveyed 5-ALA for the treatment of pediatric brain tumors, 5-ALA fluorescence was useful in 2 of 8 cases of MB (25%) [21]. Eicker et al. reported the application of 5-ALA in spinal cord tumor surgery, in which one patient had spinal cord metastasis of MB [22].

Roth et al. reported their experience in the use of 5-ALA in pediatric brain tumors. They found that aside from the GBM, 5-ALA showed a limited fluorescence amongst pediatric brain tumors, including MB [23]. One possible reason may be that differences in the mitochondrial enzyme ferrochelatase, which is thought to be downregulated in GBM cells, thus upgraded the level of protoporphyrin IX emits the fluorescence [24].

Although 5-ALA has been approved for use in countries such as Europe, Asia, and the United States. However, no approval for clinical use of 5-ALA has been obtained in China.

FS is a dye that can accumulate in the area of blood brain barrier (BBB) breakdown, presenting a major excitation wavelength peak ranging from 460 to 500 nm, and a major green emission peak fluorescent radiation ranging from 540 to 690 nm [6]. The intact BBB prevents the uptake of FS, however, the existence of a tumor can impair the BBB. It provides a good visualization of the tumor. FS is firstly applied to aid in the identification of several brain tumor types in 1948 [25]. Since then, literature reports on the application of FS in tumors have gradually increased. FS has been reported to provide significant advantages in the extent of resection for HGGs [26–28], including recurrent glioblastomas [29]. It is usually effective for the malignances which were significantly enhanced in preoperative MRI scans, such as metastatic tumors, and lymphoma [12, 30, 31]. Studies reported that FS is a feasible technique for deep-seated malignant brain tumors [12, 32]. Similar results have been reported in the spinal cord [33]. FS is also of equal value in the surgical treatment of benign tumors [34], and facilitates guided sampling in non-enhancing gliomas [9].

However, since the role of the FS depends on the destruction of the BBB, this also affects its reliability. We found that, as the operation progressed, normal brain tissue shrinkage and hemorrhage could cause obvious background interference to fluorescence imaging, making it difficult to confirm tumor boundaries solely by fluorescence.

But to the best of my knowledge, the use of FS in the surgery of MB has been reported only in one case [10]. Alysa Almojuela et al. reported the first application of FS in a 5-year-old female diagnosed as MB. But there are no more studies published about FS utilized to aid in the resection of MB. Our research shows evidence of the safety and efficiency of FS in the resection of MB. The FS-guided group can find stable fluorescence in the operation, even if the tiny satellite lesion is localized. We considered the use of FS as a promising surgical tool.

Since we started FS surgery in 2018, the number of surgical cases is still limited. And the limitation of pathologic classification in our hospital weakened the research. In terms of the benefit of FS in the protection of CNS function, our result did not provide a positive outcome. That may be due to the sample size of this research. The incidence of several symptoms was reduced in the FS group, although there was no significant difference compared with the NFS group. But we believe that there may be different results later when the sample size can be expanded.

## Conclusions

Fluorescein sodium is a safe and effective adjunct in the resection of medulloblastoma. The fluorescence in the operation is stable. Fluorescein sodium has a good potential application value in surgery, and further research is needed in the future.

## Data Availability

The data during and/or analyzed during the current study are available from the corresponding author.
